# Diagnostic accuracy, sensitivity, and specificity of CT pulmonary artery to aorta diameter ratio in screening for pulmonary hypertension in end-stage COPD patients

**DOI:** 10.3325/cmj.2021.62.446

**Published:** 2021-10

**Authors:** Kristina Gašparović, Gordana Pavliša, Maja Hrabak Paar, Marija Brestovac, Martina Lovrić Benčić, Jadranka Šeparović Hanževački, Davor Miličić, Miroslav Samaržija, Josip Juras

**Affiliations:** 1 Department of Cardiovascular Diseases, University Hospital Center Zagreb, Zagreb, Croatia; 2 Department of Respiratory Medicine, University Hospital Center Zagreb, University of Zagreb School of Medicine, Zagreb, Croatia; 3 Department of Radiology, University Hospital Center Zagreb, University of Zagreb School of Medicine, Zagreb, Croatia; 4 Department of Obstetrics and Gynecology, University Hospital Center Zagreb, University of Zagreb School of Medicine, Zagreb, Croatia

## Abstract

**Aim:**

To determine the diagnostic accuracy of pulmonary artery to aorta ratio in screening for pulmonary hypertension in advanced chronic obstructive pulmonary disease (COPD) patients.

**Methods:**

A prospective, diagnostic study was conducted in University Hospital Center Zagreb between January 2015 and March 2018. The study enrolled 100 patients who consecutively underwent chest computed tomography (CT), echocardiographic exam, and right heart catheterization. Two independent observers measured pulmonary artery and ascending aorta diameters. The correlation between the ratio and mean pulmonary artery pressure, measured invasively, was assessed. Patients with echocardiographic signs of moderate systolic or diastolic left ventricular dysfunction were excluded (n = 44).

**Results:**

Sixty-six patients (55.5% men), with a median age of 61, were identified. Median forced expiratory volume during the first second (FEV1) was 34 ± 12, FEV1/forced vital capacity <0.70. Patients with and without pulmonary hypertension had pulmonary artery diameter of 36 ± 7 mm and 27 ± 4.6 mm, respectively (*P* < 0.001). Median pulmonary artery/aorta (PA/A) ratios for patients with and without pulmonary hypertension were 1.05 and 0.81, respectively (*P* < 0.001). PA/A ratio above 0.95 was an independent predictor of pulmonary hypertension with a specificity of 100% and a sensitivity of 74.51% (area under the curve = 0.882; standard error = 0.041; *P* < 0.001).

**Conclusion:**

PA/A ratio as measured on chest CT images can be used as a screening tool instead of echocardiography.

In patients suffering from late-stage chronic obstructive pulmonary disease (COPD), pulmonary vasculature disease is a predictor of COPD progression and exacerbation. The association may be explained by the resultant pulmonary arterial hypertension (1-3). As pulmonary hypertension develops throughout a patient’s lifetime, varying degrees of subclinical and clinical pulmonary vascular pathology are found. In a subset of patients with advanced COPD, pulmonary hypertension development is associated with a decreased functional status and an increased mortality (4,5). Furthermore, transplant-free patients with pulmonary hypertension have a markedly reduced survival rate. In some patient groups, the timing of lung transplantation is determined by elevated pressures in the pulmonary circulation (6). For these reasons, detection of pulmonary hypertension is vital as it influences the decisions on medical and surgical treatment.

Pulmonary hypertension remains a diagnostic challenge due to its non-specific symptoms: malaise, dyspnea on exertion, and fatigue, along with many other pulmonary disease presentations. Pulmonary hypertension is ultimately treated by surgery: single lung transplant, bilateral lung transplant, or heart and lung transplant (6). However, lung volume reduction surgery can be contraindicated in patients with advanced lung emphysema (7). Hence, adequate treatment requires early and precise detection of pulmonary hypertension (8).

Currently, right heart catheterization (RHC) is the gold standard in pulmonary hypertension verification (9,10), but this invasive method carries the risk of complications (ie, malignant arrhythmias, heart chamber perforation, pericardial effusion).

A non-invasive, widely available method of measurement of the right heart and pulmonary artery pressures is echocardiography; it is validated and well-established in clinical practice for pulmonary hypertension detection. However, in COPD patients it is often burdened with inadequate acoustic windows due to hyperinflation (11).

Late-stage COPD patients require routine follow-up computed tomography (CT) scanning. CT enables pulmonary artery (PA) and aorta (A) diameter measurement and PA/A ratio determination on a single scan. Hence, in these patients the PA/A ratio could be used as a valuable diagnostic tool for pulmonary hypertension assessment, obviating the need for RHC. On the other hand, patients with a high PA/A score could benefit from a timely referral for invasive diagnostic assessment. Recent studies suggested that PA/A ratio could be a relevant marker of PH in COPD patients (3,12,13). However, only a few studies have evaluated PA diameter in end-stage COPD patients (14,15). Thus, the aim of this study was to determine the cut-off value of PA/A ratio measured on CT that could be applied as a screening tool for pulmonary hypertension in end-stage COPD patients.

## Patients and methods

### Patients

This prospective, diagnostic study enrolled all patients diagnosed with COPD in the group C or D according to the GOLD standards. All patients were evaluated for lung transplantation from January 2015 to March 2018 in the University Hospital Center Zagreb. The patients' data obtained by clinical assessment, RHC, echocardiography (ECHO), and CT were collected within a six-month period. The inclusion criteria included stable disease with no patient in acute exacerbation during CT, ECHO examination, or RHC. We excluded the patients with another pulmonary pathology (asthma, pneumonia, tumor, idiopathic pulmonary fibrosis), those with systolic dysfunction of the left ventricle, those with greater than mild diastolic dysfunction or any valvular pathology, and non-compliant patients.

**Figure 1:**
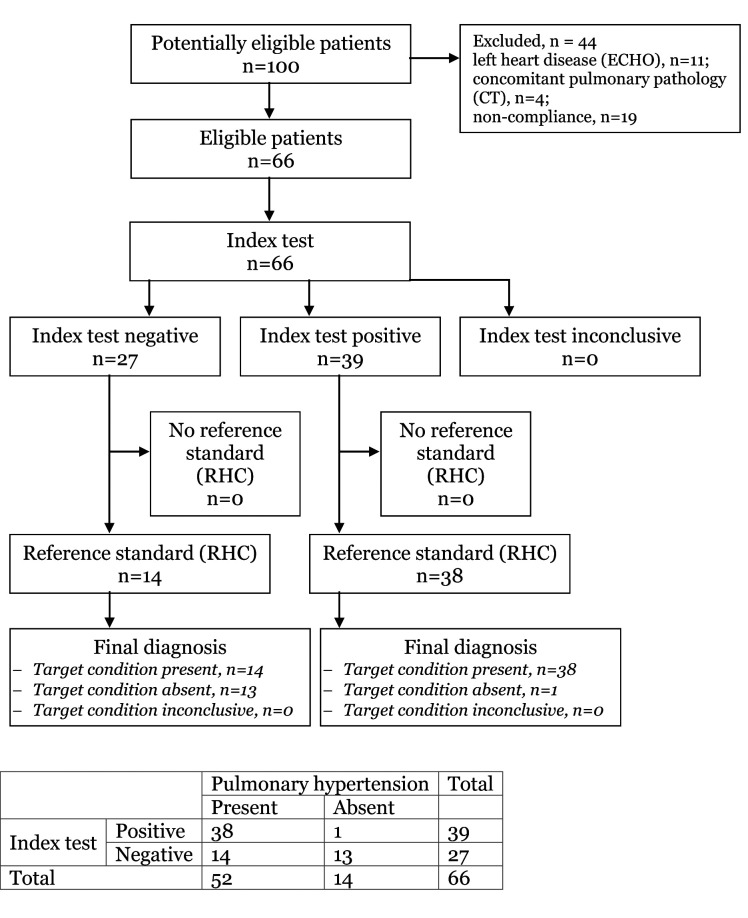
Study flowchart. ECHO – echocardiography; CT – computed tomography; RHC – right heart catheterization.

One hundred patients with verified end-stage COPD based on spirometry findings were consecutively enrolled (Figure 1). We presumed the prevalence of pulmonary hypertension within the studied population to be 75%, and the sensitivity for target condition to be 80%, which allows the study power of 90%. After ECHO examination and CT, 34 patients were excluded: 11 because of left heart disease verified by ECHO, 4 because of concomitant pulmonary pathology verified on CT, and 19 were lost to follow-up because of non-compliance. In total, 66 patients continued the study, with data sets of RHC data, ECHO parameters, and CT findings. Based on mPAP assessed by RHC, the study population was divided into two groups: a control group with mPAP<25 mm Hg and a PH group with mPAP≥25 mm Hg. PA and A diameters were measured on CT in both study groups. Indeterminate results would have been considered as negative, but there was none. The study was approved by the Ethics Committee of UHC Zagreb (02/21/JG).

### Demographic and clinical assessment

Demographic and clinical variables obtained were sex, age, body mass index (BMI), and smoking habits. Functional status was derived from the BODE index including 6-minute walking test (6-MWT). All patients completed the COPD Assessment Test (CAT) questionnaire and modified Medical Research Council (mMRC) dyspnea scale.

Spirometry revealed information regarding forced expiratory volume in the first second of exhalation (FEV1); predicted values of FEV1, predicted values of total lung capacity, and functional vital capacity (FVC). A patient with COPD was defined as one with post-bronchodilator spirometry FEV1/FVC ratio of less than 70%.

### Echocardiographic assessment

ECHO examination included the assessment of the systolic and diastolic left ventricular function. Pulmonary hypertension values were obtained by calculating the maximum velocity of the jet of tricuspid insufficiency using the Bernoulli equation and adding the presumed pressure in the right atrium (regarding flow in the inferior vena cava).

### Right heart catheterization 

RHC was carried out by three different specialists, and the data collected were mean mPAP, systolic arterial pressure, and diastolic pulmonary arterial pressure, cardiac output, cardiac index, and pulmonary capillary wedge pressure. Pulmonary hypertension was defined as mPAP≥25 mm Hg.

### Computed tomography scan

**Figure 2:**
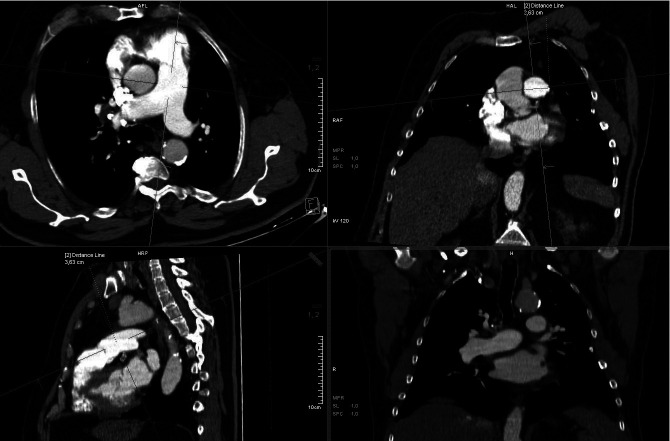
Measurement of the pulmonary artery and aorta width on the same slice of the computed tomography scan.

Pulmonary CT angiography was performed using a 40-detector row CT scanner (SOMATOM Sensation 40, Siemens Medical Solutions, Erlangen, Germany) with breath-hold technique. CT scans were analyzed by two radiology specialists blinded to patients’ clinical history. The measurements were performed at the level of the bifurcation of the main PA, and the aorta diameter was measured at the same slice (Figure 2). Both radiologists performed precise and accurate measurements, with no inter-rater bias (kappa test performed, K_1_ = 0.874, K_2_ = 0.836, respectively). The patients with abnormal pulmonary CT findings were excluded from the study (interstitial lung disease, bronchiectasis, any mass or fibrosis).

### Statistical analysis

Demographic and clinical characteristics were summarized as medians and 25th and 75th percentile for continuous variables and as the number and percentage for categorical variables. The Shapiro-Wilk test was used to test the normality of distribution. The Fischer exact and Fischer-Freeman-Halton exact tests were used to assess the differences in categorical variables. The Mann-Whitney U test was implemented to analyze the difference between PA and aorta dimensions in patients with and without pulmonary hypertension. Changes in the specificity and sensitivity of PA/A ratio for the detection of pulmonary hypertension were analyzed by receiver operating characteristic (ROC) curve. Youden index was used to determine the cut/off value of PA/A ratio with the best specificity/sensitivity relationship for pulmonary hypertension prediction. *P* values lower than 0.05 were considered statistically significant. Data were analyzed with MedCalc Statistical Software, version 18.2.1 (MedCalc Software bvba, Ostend, Belgium).

## Results

The baseline clinical characteristics are shown in Table 1. RHC revealed pulmonary hypertension in 52 patients, and excluded pulmonary hypertension in 14 patients (control group). The median age of controls was 58.5 and that of the pulmonary hypertension group was 60 years. There were 30 women included (45.5%).

**Table 1:** Differences in sociodemographic and clinical characteristics of patients with pulmonary hypertension and control group*

Variable	Group	Median	25-75 percentile	P^‡^
Age (years)*	control	58.50	48.00-66.00	0.278
	PH	60.00	39.00-75.00	
Smoking years (n)	control	20.00	0.00-35.75	0.651
	PH	30.00	20.00-30.00	
Pack per year (n)	control	35.00	0.00-52.50	0.788
	PH	30.00	20.00-40.00	
BMI (kg/m^2^)	control	27.53	26.42-30.10	0.068
	PH	26.32	23.60-28.25	
BSA (m^2^)	control	1.94	1.90-2.02	0.245
	PH	1.90	1.77-2.02	
Exacerbations in one year (n)	control	3.00	2.00-3.00	0.083
	PH	3.00	3.00-3.75	
CAT	control	28.00	24.50-31.25	0.894
	PH	28.50	25.00-32.75	
FVC (%)	control	59.00	40.05-78.00	0.784
	PH	57.20	47.43-72.03	
FEV1 (%)	control	32.50	20.43-50.00	0.551
	PH	33.70	22.20-46.08	
FEV1/FVC	control	45.30	35.20-65.00	0.678
	PH	49.95	39.00-61.00	
DLCO	control	37.50	32.50-55.00	0.090
	PH	50.00	40.00-65.00	
6-MWT	control	240.00	180.00-370.00	0.052
	PH	182.50	125.00-267.50	
BODE	control	5.50	2.00-6.25	0.006
	PH	6.00	4.25-7.00	

*Abbreviations: PH – pulmonary hypertension; BMI – body mass index; BSA – body surface area; CAT – COPD assessment test; FVC – forced volume vital capacity; FEV1 – forced expiratory volume in 1st second; FEV1/FVC – the Tiffeneau-Pinelli index; DLCO – diffusing capacity for carbon monoxide; 6-MWT six-minute walking test; BODE – body mass index, airflow obstruction, dyspnea, and exercise.

‡Mann-Whitney U test.

Median (standard deviation [SD]) FEV1% was 32.5 (20.4-50) in the control group and 33.7 (22.2–46.0) in the pulmonary hypertension group, with no significant difference between the groups. The median mPAP in the control group was 23 (21.7–24) mmHg and that in the pulmonary hypertension group was 40.5 (34.0-47.75) mmHg.

Our study group consisted of 52 patients (78.8%) with pulmonary hypertension; 23 were women (44.2%). The PH group and controls did not significantly differ in age, sex, BMI, FEV1, and FEV1/FVC.

Six-minute walking test result was lower in the pulmonary hypertension group, but the difference did not reach significance (*P* = 0.052). The groups did not differ in the CAT questionnaire results. Patients with pulmonary hypertension had a significantly higher BODE index (FEV1, 6-MWT, MMRC dyspnea scale, BMI) than controls: 6.0 (4.3-7.0) vs 5.5 (2.0-6.3) (*P* = 0.006).

**Figure 3:**
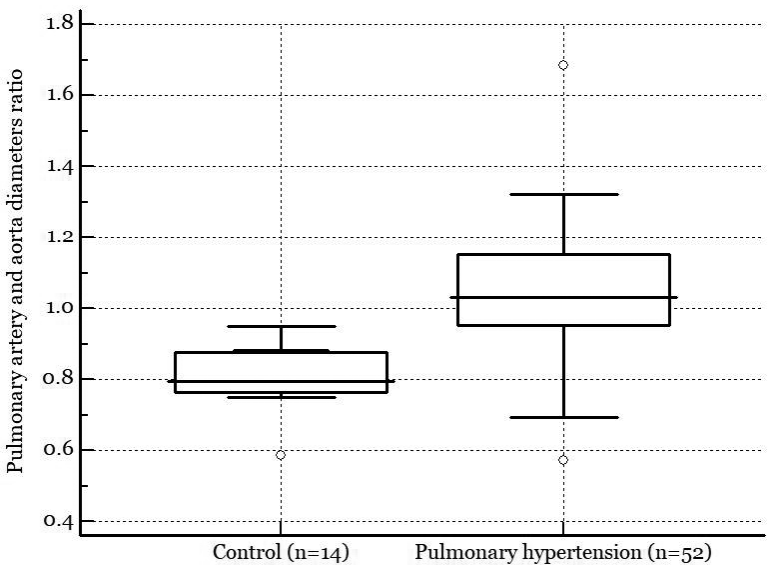
Higher pulmonary artery/aorta diameters ratio in pulmonary hypertension group compared with the control group.

Patients with PH had a higher median PA diameter than controls (36.10 [22-63] mm vs 27.50 [17.8-38] mm, *P* < 0.001) and a significantly higher median PA/A ratio (1.06 vs 0.80, *P* < 0.001) (Table 2, Figure 3).

**Table 2:** Computed tomography and right heart catheterization measurements in the PH and control group

Variable	Group	Median	25-75 percentile	P†
PA (mm)	control	27.50	24.75-29.00	<0.001
	PH	36.10	31.05-40.75	
Aorta (mm)	control	32.50	30.75-36.00	0.387
	PH	34.00	31.40-37.85	
PA/A	control	0.80	0.76-0.89	<0.001
	PH	1.06	0.97-1.17	
Vena cava superior (mmHg)	control	9.50	5.00-10.50	0.028
	PH	12.00	9.00-15.00	
Right atrium pressure (mmHg)	control	9.00	5.75-11.75	0.024
	PH	12.00	10.00-14.75	
RVsis (mmHg)	control	34.00	33.00-37.00	<0.001
	PH	59.05	51.25-76.50	
RVd (mmHg)	control	2.00	1.00-5.00	0.004
	PH	5.00	2.00-7.00	
RVm (mmHg)	control	15.50	13.75-16.25	<0.001
	PH	26.00	21.50-35.75	
PAs (mmHg)	control	35.00	33.00-37.00	<0.001
	PH	59.50	53.25-79.50	
PAd (mmHg)	control	14.00	12.00-16.00	0.001
	PH	28.00	24.25-33.00	
PAm (mmHg)	control	23.00	21.75-24.00	<0.001
	PH	40.50	34.00-47.75	
PCWP (mmHg)	control	10.00	8.00-11.25	0.037
	PH	11.00	9.00-13.00	
Cardiac output (L/min)	control	4.88	4.45-5.74	0.330
	PH	5.46	4.53-5.80	
Cardiac index (L/min/m^2^)	Control	2.79	2.28-2.95	0.742
	PH	2.70	2.50-3.09	
PVR (dynes/s/cm^5^)	control	220.70	189.75-239.80	<0.001
	PH	461.17	360.25-568.43	
SVR (dynes/s/cm^5^)	control	1472.50	1309.20-1657.50	0.475
	PH	1376.70	1210.75-1667.25	
PVR/SVR	control	0.14	0.12-0.18	0.001
	PH	0.35	0.24-0.41	
EF (%)	control	60.00	60.00-65.00	0.907
	PH	60.00	60.00-65.00	

*Abbreviations: PH – pulmonary hypertension; PA – pulmonary artery diameter; A – aortal diameter; RVsis – systolic pressure in the right ventricle; RVd – diastolic pressure in the right ventricle; RVm – mean pressure in the right ventricle; PAs – systolic pressure in the pulmonary artery; PAd – diastolic pressure in the pulmonary artery; PAm – mean pressure in the pulmonary artery; PCWP – pulmonary capillary wedge pressure; PVR – pulmonary vascular resistance; SVR – systemic vascular resistance; EF – ejection fraction of the left ventricle.

†Mann-Whitney U test.

**Figure 4:**
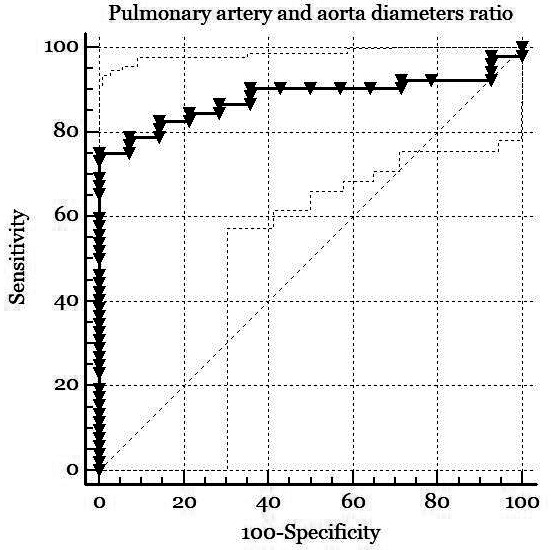
Receiver operating characteristic (ROC) analysis showing pulmonary artery/aorta (PA/A) diameter as an excellent classifier for the presence of pulmonary hypertension. Area under the curve = 0.882; standard error = 0.042; z = 9.266; *P* < 0.001, sensitivity 74.51% (95% CI 61.1-86.0); specificity 92.86% (95% CI 66.1-99.8) at PA/A diameter ratio of ≥0.95; accuracy of test = 82.97% (95% CI 71.71-91.10).

ROC analysis showed an excellent area under the curve when PA/A ratio was explored as a diagnostic tool for the presence of pulmonary hypertension (Figure 4). The highest Youden index (J = 0.750) gave us a cut-off value of ≥0.95 for the presence of PH, with a sensitivity of 74.51% (95% CI 61.1-86.0), specificity of 92.86% (95% CI 66.1-99.8) (AUC = 0.882; SE = 0.041; z = 9.266; *P* < 0.001), and accuracy of 82.97% (95% CI 71.71-91.10). The odds ratio for having PA/A ratio above 0.95 in late-stage COPD patients was 42.25 (95% CI 5.00-357.10; z = 3.438; *P* = 0.001).

**Figure 5:**
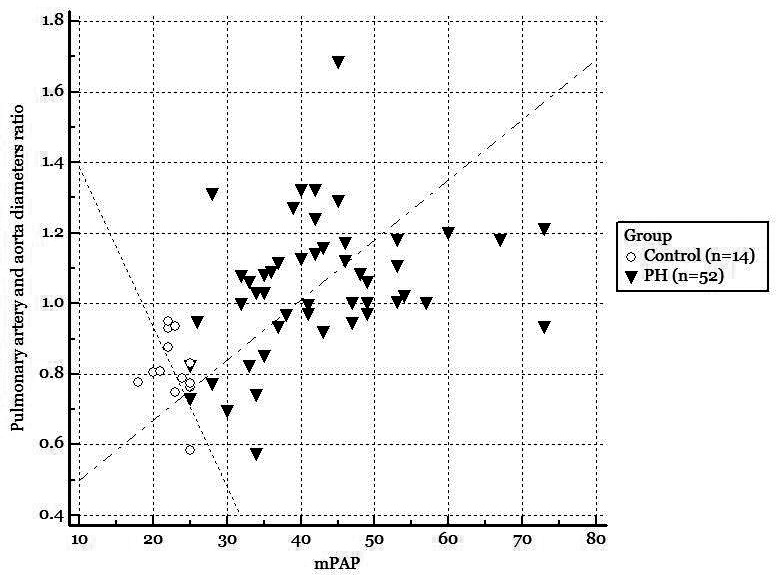
Significantly higher correlation coefficients of pulmonary artery/aorta (PA/A) and mean pulmonary artery pressure (mPAP) in the pulmonary hypertension (PH) croup compared with the control group.

In the pulmonary hypertension group, PA/A moderately positively correlated with mPAP (rho = 0.381, *P* = 0.006). In the control group, the correlation coefficient was not significant (rho = -0.402, *P* = 0.154). The difference between the two correlation coefficients was significant (z = 2.480, *P* = 0.013), confirming the importance of the PA/A ratio as a marker of pulmonary hypertension. The difference in correlation coefficients of PA/A ratio and mPAP was assessed by z-statistics (Figure 5). No adverse events were reported during CT scanning and RHC.

## Discussion

In our study, PA/A ratio as measured on CT was associated with pulmonary hypertension in patients suffering from late-stage COPD. PA/A≥0.95 showed a significant diagnostic accuracy for predicting elevated mPAP on RHC, with a sensitivity of 74.5% and specificity of 92.86%. All patients with PA/A≥0.95 had PH confirmed invasively, while 23% of patients with verified PH did not have PA/A≥0.95.

In the PH group, PA/A significantly positively correlated with mPAP, while no such correlation was found in the control group. The significant difference between these two correlation coefficients indicates the potential of PA/A ratio as a new diagnostic parameter.

Pulmonary artery diameter measurement is a relatively new method in clinical practice. This method can help us to identify the patients with pulmonary hypertension who are at risk of increased mortality (16,17) and COPD exacerbation.

Iliaz et al (18) showed, on 156 patients hospitalized for COPD exacerbations, that PA/A ratio positively correlated with the number of hospitalizations due to COPD in one year. In another study, PA/A>1 was significantly related to the number of exacerbations (19). However, this study made no comparison with the level of pulmonary hypertension (19).

Terzikan et al (20), in 2197 participants from the population-based Rotterdam Study, demonstrated no association between 1-SD increase in PA/A and mortality in the general population, but observed this association in moderate-to-severe COPD patients. Previous studies reported an association between PA/A ratio >1 and pulmonary hypertension in COPD, but most of them included patients with various lung pathologies (21-23).

An early pulmonary hypertension diagnosis in COPD patients is important because an adequate approach may improve the patient’s quality of life and survival (24,25). This is in part due to the higher complication risk and increased morbidity and mortality in patients with pulmonary hypertension (6,26).

Ersoy et al (27) observed a correlation between echocardiography-guided measurements of PAP and PA/A>1.0 in patients with acute exacerbation of COPD. However, no significant association between higher PA/A ratio and increased mortality was observed. Furthermore, an increased PA/A ratio or enlarged PA diameter on CT were found to be a useful tool for a timely and proper clinical assessment (28).

Our study involved a well-defined sample of patients with an end-stage COPD. In a similar study, Hoesein et al (15) retrospectively studied 92 patients, 32.6% of whom had pulmonary hypertension, and observed that PA/A>1 had a 50% sensitivity and 85.5% specificity in identifying pulmonary hypertension. Higher sensitivity and specificity obtained in our study may be explained by a proportionally larger number of patients with pulmonary hypertension.

Patients in advanced stages of COPD require periodic CTs, and our method of assessing pulmonary hypertension by PA/A ratio can indicate an invasive diagnostic approach. CT is a non-invasive and accessible tool in routine clinical practice, allowing us to get additional valuable information using this novel approach.

Symptoms of pulmonary hypertension are unspecific and overlap with those of COPD without pulmonary hypertension. However, the PA/A ratio can be used to guide the clinical decision concerning the use of RHC.

Our study showed that an elevated PA/A ratio and mPAP are mainly influenced by PA enlargement rather than by changes in aortic dimensions. However, some other mechanisms could also contribute to PA enlargement, such as pulmonary arterial distensibility (23) and redistribution of blood flow from capillary loss on periphery (29).

In this study, we excluded patients with left ventricular systolic dysfunction and greater than mild diastolic dysfunction. Some studies found a correlation between left heart disease and PA size (30). Echocardiography is widely used as a screening tool for estimating pulmonary hypertension (31-34). However, the presence of a hyper-inflated thorax in COPD patients makes the examination impossible to perform in about 40% of the patients (21). We were able to measure systolic PAP non-invasively in 69% of patients, while in one third of patients echocardiography was insufficient in PH detection.

The limitations of the study include a relatively small sample size. However, the high number of patients with proven PH makes the assessment of pulmonary vasculature reliable. All patients were thoroughly examined by echocardiography, and all patients with a reduced ejection fraction, a greater than mild diastolic dysfunction, and valvular pathology were excluded. There is concern regarding the study applicability since 44/100 patients were not enrolled in the study (left heart disease [ECHO], n = 11; concomitant pulmonary pathology [CT], n = 4; non-compliance, n = 19). Additionally, due to the small number of published studies on this subject there is no reference standard for PA/A ratio. The advantages of the study include no risk of bias due to flow and timing, and no inter-rater bias.

To summarize, according to our data, the PA/A ratio ≥0.95 as measured by CT could be a highly accurate tool to predict the presence of pulmonary hypertension in end-stage COPD patients. The odds ratio, due to small sample size, had a huge 95% confidence interval. Stage C and D COPD patients who had a PA/A ratio ≥0.95 had a 42 times greater probability of having pulmonary hypertension in comparison with patients with PA/A ratio <0.95, or at least a 5 times greater risk, if we take the lower value of 95% confidence interval. However, the presence of pulmonary hypertension cannot be excluded in patients with PA/A<0.95. Meta-analyses including more data are necessary to confirm the reliability and accuracy of PA/A ratio as a measure of the pulmonary hypertension risk in stage C and D COPD patients. Therefore, invasive RHC measurement remains the most important and unavoidable method for detecting pulmonary hypertension.
